# Transcriptional Profiling and Machine Learning Unveil a Concordant Biosignature of Type I Interferon-Inducible Host Response Across Nasal Swab and Pulmonary Tissue for COVID-19 Diagnosis

**DOI:** 10.3389/fimmu.2021.733171

**Published:** 2021-11-22

**Authors:** Cheng Zhang, Yi-Gang Feng, Chiwing Tam, Ning Wang, Yibin Feng

**Affiliations:** ^1^ School of Chinese Medicine, Li Ka Shing Faculty of Medicine, The University of Hong Kong, Hong Kong, Hong Kong SAR, China; ^2^ Guanghua School of Stomatology, Hospital of Stomatology, Sun Yat-Sen University, Guangzhou, China

**Keywords:** COVID-19, SARS-CoV-2, type I interferon, diagnosis, machine learning

## Abstract

**Background:**

COVID-19, caused by SARS-CoV-2 virus, is a global pandemic with high mortality and morbidity. Limited diagnostic methods hampered the infection control. Since the direct detection of virus mainly by RT-PCR may cause false-negative outcome, host response-dependent testing may serve as a complementary approach for improving COVID-19 diagnosis.

**Objective:**

Our study discovered a highly-preserved transcriptional profile of Type I interferon (IFN-I)-dependent genes for COVID-19 complementary diagnosis.

**Methods:**

Computational language R-dependent machine learning was adopted for mining highly-conserved transcriptional profile (RNA-sequencing) across heterogeneous samples infected by SARS-CoV-2 and other respiratory infections. The transcriptomics/high-throughput sequencing data were retrieved from NCBI-GEO datasets (GSE32155, GSE147507, GSE150316, GSE162835, GSE163151, GSE171668, GSE182569). Mathematical approaches for homological analysis were as follows: adjusted rand index-related similarity analysis, geometric and multi-dimensional data interpretation, UpsetR, t-distributed Stochastic Neighbor Embedding (t-SNE), and Weighted Gene Co-expression Network Analysis (WGCNA). Besides, Interferome Database was used for predicting the transcriptional factors possessing IFN-I promoter-binding sites to the key IFN-I genes for COVID-19 diagnosis.

**Results:**

In this study, we identified a highly-preserved gene module between SARS-CoV-2 infected nasal swab and postmortem lung tissue regulating IFN-I signaling for COVID-19 complementary diagnosis, in which the following 14 IFN-I-stimulated genes are highly-conserved, including BST2, IFIT1, IFIT2, IFIT3, IFITM1, ISG15, MX1, MX2, OAS1, OAS2, OAS3, OASL, RSAD2, and STAT1. The stratified severity of COVID-19 may also be identified by the transcriptional level of these 14 IFN-I genes.

**Conclusion:**

Using transcriptional and computational analysis on RNA-seq data retrieved from NCBI-GEO, we identified a highly-preserved 14-gene transcriptional profile regulating IFN-I signaling in nasal swab and postmortem lung tissue infected by SARS-CoV-2. Such a conserved biosignature involved in IFN-I-related host response may be leveraged for COVID-19 diagnosis.

## Introduction

The novel coronavirus disease 2019 (COVID-19) induced by SARS-CoV-2 infection has resulted in a sustained threat to human life and economic growth. As of September 2021, around 4.6 million SARS-CoV-2-infected deaths have been reported by WHO, showing an unprecedented challenge and need for controlling the COVID-19 pandemic. Respiratory dysfunction is the main complication of COVID-19, including diffused alveolar damage and fulminant respiratory failure (1). Notably, clinical manifestations of SARS-CoV-2 infection vary from asymptomatic to severe symptoms (2). Such a wide range of clinical features make it difficult to establish a highly-conserved diagnostic profile of COVID-19. Although scientists have made great progress on COVID-19 management, progress in the viral diagnosis seems to be inferior to the development of therapy and prevention for COVID-19. Till now, diagnostic measurement of COVID-19 mainly relies on the reverse transcription quantitative polymerase chain reaction (RT-PCR) due to excellent sensitivity and specificity for detecting SARS-CoV-2 (3). However, using RT-PCR alone may yield false-negative results due to fluctuated viral loads and evolution (4). This adverse situation is detrimental for hampering COVID-19 outbreak. Improving the accuracy of viral testing remains urgent demand. Apart from the direct recognition for SARS-CoV-2, deciphering the host response, especially the virus-related fluctuated genomic profile, may be pivotal for serving as a supplementary approach for COVID-19 diagnosis.

For the genetic profile of host response for COVID-19, multiple studies have reported various characteristics of immune/inflammatory actions in response to COVID-19. For example, activation of immune cells was observed in lung and bronchoalveolar lavage fluid (5). Cytokine or chemokines-related host inflammatory responses were involved in SARS-CoV-2 infected bronchoalveolar lavage and peripheral blood mononuclear cells (PBMCs) (6). Besides, COVID-19 progression is driven by the populations of myeloid-lineage cells with distinct inflammatory transcriptional features in blood, lung, and airway (7). These findings suggested that identification of a specific genomic profile of host response may be served as a supplementary method for COVID-19 diagnosis. For discovering COVID-19 related genomic trait for diagnosis, it may have three features. 1) It is representative for COVID-19 diagnosis and different from other respiratory diseases (such as Influenza, Measles, and Respiratory Syncytial Viral). 2) The specific gene expression feature detected in body substance is prefer to non-invasively collect for diagnosis, such as nasal swab. Of note, although nasal swab was extensively used for direct virus detection by RT-PCR, genomic profile of specific genes in nasal fluid may also unveil the feature of host response to COVID-19, such a molecular feature may be used as a biological basis for reducing the false-negative diagnosis. 3) Since lung is the main-affected organ in COVID-19, the diagnostic feature in extrapulmonary substances (e.g., nasal fluid) may be highly-preserved with that in pulmonary tissue. Recently, scientists have engaged in discovering the COVID-19 immune landscape. Dramatic transcriptomic changes were detected in virus-positive cells in severity-dependent manner. These differential genes were enriched in specific pathways, including “response to virus” and “response to type I interferon” (8). SARS-CoV-2 induced transcriptomic changes in the peripheral blood is varied with those detected in other respiratory infections, including interferon-driven genes (9). Besides, Nature News announced the top 10 awesome science discoveries in 2020, including “Interferon deficiency can lead to severe COVID-19, especially the IFN-I” (10, 11). These studies suggested the strong correlation between “Type I interferon and COVID-19”. As a first-line innate host defense mechanism, human Type I interferons (IFN-I) are a large family of interferon proteins (IFNα and IFNβ, etc.) that regulate the immune system, such as the inhibition of virus proliferation and transmission (12, 13). Besides, it is documented that robust cellular secretion of IFN-I is indispensable for suppressing SARS-CoV-2 replication (14). Although anti-SARS-CoV-2 effect of IFN-I has been widely reported, the potential diagnostic role of IFN-I for COVID-19 is under investigation. Recently, several reports have discussed IFN-I-related host defense in COVID-19 (15, 16). These valuable studies may potentially indicate molecular clues that SARS-CoV-2 affected IFN-I-dependent gene profile may be used as a complementary diagnosis of COVID-19. Hereby, we listed the evidenced descriptions and our own postulations are as below: 1) It was reported that high density of receptors of ACE2 (an enter-receptor of SARS-CoV-2) causes the high SARS-CoV-2 viral load in nasopharyngeal fluid in the initial stage of COVID-19 but lowered during sustained viral infection in nasal swab (17). It may suggest that testing SARS-CoV-2 viral load alone in nasal swab is defective because of the potentially low viral content and evolution. 2) Nasal swab samples remain to be the main source of RT-PCR-based SARS-CoV-2 nucleic acids testing due to the high viral load in nasopharyngeal fluid before severe COVID-19 (18). Nasal swab remains to be an easily handled and non-invasive reliable testing approach worldwide (19). Additionally, the time window for virus detection *via* nasal swab lasts around 4 weeks and peaks in the second week from the onset of infection (20). The 4-week detectable cycle of genetic profile is not too transient and enables us to timely conduct COVID-19 diagnosis. These reports revealed that understanding the genetic host response in nasal swab may be useful for improving COVID-19 diagnosis. 3) In SARS-CoV-2 infected tissues, lung is the most vulnerable organ responsible for the mortality of COVID-19, which includes upper respiratory tract infections (in early stage) and acute respiratory distress syndrome (in late-stage) (21), suggesting that molecular profile in pulmonary host immune response is vital for COVID-19 diagnosis. It is critical to clarify the molecular correlation between nasal swab and lung tissue in response to COVID-19, because COVID-19 diagnosis by nasal swab may still be the common way due to the non-invasive collection and relatively higher virus load. 4) Interferon is one of the regulators of ACE2 receptor (22). Additionally, host IFN-I possesses high sensitivity to both SARS-CoV-2 virus and ACE2 receptors, especially in COVID-19 patients with asymptomatic manifestation (23), suggesting that understanding the genetic signature of IFN-I-associated genes in SARS-CoV-2 infection may serve as indicators of COVID-19 diagnosis. 5) Recent reports suggested that detection of IFN-I gene expression may be of great significance to measure the severity of COVID-19 (21), indicating that varied IFN-I-related gene profile may stratify the severity of COVID-19. Taken together, these five clues provide prerequisites for mining a molecular feature of IFN-I-related host response for the diagnosis of COVID-19 and its severity.

Global scientists have conducted a series of clinical trials not only for COVID-19 diagnosis, but also the comparative analysis between COVID-19 and other respiratory infectious diseases, including SARS and MERS (24). Generally, these reported outcomes have two features as follows: 1) Generally, the results are independently generated from homogeneous samples. Due to the complexity of body tissues, the correlation of genetic feature among heterogeneous tissues needs to clarify. A highly-preserved profile has diagnostic potential for COVID-19. 2) Gene relationship in homogeneous or heterogeneous samples are usually determined *via* geometric distance (commonly by differential expression). Measuring adjacency-related similarity in a scale-free network from heterogeneous samples may make the results more biologically significant, since real-world networks are often claimed to be scale free (25). Based on these ideas, our study aims to identify a common diagnostic host characteristic from nasal swabs and lung tissues that can supplement the diagnostic strategy of COVID-19. Highly-conserved functional genes modules are commonly related to the central characteristic of a disease (26). Combined with RT-PCR-based virus detection, the specific profile of host response may provide additional information for distinguishing COVID-19 from other respiratory diseases, in which we focused on IFN-I related gene modules. Herein, eight independent RNA-sequencing datasets were retrieved from NCBI-GEO, including three nasal swabs (GSE163151, GSE162835, and GSE182569-nasal swab part); two lung tissues (GSE171668 and GSE150316), one lung bronchioalveolar fluid (GSE182569—lung bronchioalveolar fluid part), one lung bronchial epithelial cells (GSE147507), and one lung bronchoalveolar carcinoma cells (GSE32155).

For the analytic methods, we adopted computational language R-based unsupervised analysis for clarifying the genetic polymorphisms and highly-preserved functional gene modules. As an analytical machine-learning language in computer science, R language has a wide variety of statistical techniques for life science, including WGCNA, homological and high-dimensional multivariate analyses (27). Breakthroughs in interdisciplinary technologies between computer technology and life science may permit a holistic view of transcriptomic profile and delineate gene modules with pathophysiologic relevance in COVID-19 diagnosis. In this study, we made an attempt to identify the highly-preserved genes/modules regulating IFN-I signaling in SARS-CoV-2-infected heterogeneous samples (nasal swab and lung tissue) using transcriptional and machine-learning analysis. This intent is to complement current diagnostic strategy of COVID-19. Accurate SARS-CoV-2 detection is a significant starting point to counter COVID-19 pandemic.

## Methods

### Data Acquisition, Normalization, and Filtering

Seven independent clinical RNA-seq datasets (*Homo sapiens*) were retrieved from the Gene Expression Omnibus (GEO) database using RStudio (R Inc, USA) with R function “GEOquery”, including GSE163151 (nasal swabs-COVID-19, Respiratory syncytial viral, Influenza A, Influenza B, GSE162835 (nasal swabs-COVID-19), GSE182569 (nasal swabs or lung bronchioalveolar fluid-COVID-19), GSE171668 (lung tissue-COVID-19), GSE150316 (lung tissue-COVID-19), GSE147507 (lung bronchial epithelial cells-COVID-19), and GSE32155 (lung bronchoalveolar carcinoma cells-Measles). The genome-wide sequencing platforms were Illumina NovaSeq 6000 (GSE163151, GSE162835), Illumina NextSeq 500 (GSE171668, GSE150316), Ion Torrent S5 (GSE182569), Illumina Nextseq 500 (GSE147507), and Aglient human genome microarray 014850 (GSE32155), respectively. DESeq2-based data normalization was performed for a variance-stabilizing transformation by R function “DESeq2”. Meanwhile, outliers in datasets were eliminated by unsupervised hierarchical clustering in a dendrogram using R function “hclust ()”, and homogeneous data were grouped in a consensus branch site. Finally, normalized RNA-sequencing data without outliers were used for subsequent data filtering. As for GSE163151, this dataset contains samples of nasal swab with several respiratory infections: non-COVID-19 (n = 91), COVID-19 (n = 73), Influenza A (n = 55), Influenza B (n = 15), and Respiratory Syncytial Viral (RSV, n = 8). For GSE171668 (n = 16) and GSE150316 (n = 5), these datasets contain postmortem lung tissues from COVID-19 patients. GSE147507 (n = 3) stands for SARS-CoV-2-infected lung bronchial epithelial cells. For GSE162835, it contains nasal swabs in COVID-19 (n = 37). For GSE32155, nasal swabs infected with Measles were further used for differential diagnosis (n = 3) For GSE182569, it includes SARS-CoV-2 infected samples from nasal swab (n = 3) and lung bronchioalveolar fluid (n = 3). Among these datasets, both GSE171668 (lung-COVID-19) and GSE163151 (nasal swab-COVID-19) were used for WGCNA analysis (**Supplementary Material 3**), while others were adopted for the 14 gene-related proof-of-concept study. The normalized gene expressions without batch effect were applied for the validation of gene profile (**Supplementary Material 4**—COVID-19 vs COVID-19; **Supplementary Material 5**—COVID-19 vs other respiratory infectious diseases).

For data filtering, those gene expression variance greater than 90% of the whole genome were adopted as the influenced and dominant genes. The missing values or zero-variance expression genes with correlative strength less than 0.1 were removed (minRelativeWeight = 0.1 in R). The function “apply()” in R was used for calculating the gene expression variance. Finally, the filtered data were further utilized for analyzing the consensus of gene profile with machine learning approaches. The R codes are available in **Supplementary Material 10**.

### Construction of Co-Expression Modules

The consensus gene modules of nasopharyngeal pulmonary specimens were constructed by WGCNA analysis. In brief, the pairwise similarity of co-expression matrixes was discerned by the coefficient resulted from Pearson’s correlation analysis for the whole genome (equation: sij = cor(i, j)). A weighted adjacency matrix was constructed for improving the consensus similarity of gene modules by the equation as follows: aij = | (1 + cor(i, j))/2 |β, in which “aij” pointed to the value of adjacency for evaluating the strength of weighted connectivity. β was the soft-thresholding power resulted from a scale-free topological analysis by the R function “pickSoftThreshold ()” from WGCNA package. Then, a topological overlap matrix (TOM) was established in terms of the adjacency matrix, which in-turn converted into a dissimilarity TOM. Afterwards, gene clustering dendrogram consisted of genes with hierarchical clustering and gene modules with various colors upon adjacency-based dissimilarity. All the modules were constructed by clustering the close-distance modules according to the resulted Module Eigengene (ME) values. MEs were based on the first principal component of gene modules by PCA analysis using R function “signedKME()” from WGCNA package. The higher absolute value of ME represented the more intense relationship between genes and their corresponding modules.

### Relevance Analysis of Co-Expression Modules

For determining the reliability and underlying correlations among constructed gene modules, module eigengenes were recruited to assign expression to gene modules for association study. TOM-based topological overlap plot was performed in accordance with the dissimilarity of gene expressions, which was visualized by R function “TOMplot()” in WGCNA package. In a topological overlap plot, rows and columns were pointed to genes. While at the top and left side were the colors related to gene modules. Darker or lighter blocks in the figure represented the low or high correlation, respectively. Besides, in terms of ME value, the module interactions were multi-dimensionally observed by both 3D scatter plot (R function “ScatterPlot3D”) and t-SNE analysis (R function “Rtsne”), respectively. As an intersective algorithm, R function UpsetR (R function “UpsetR”) was used to detect the potential overlapping targets among modules for evaluating the reliability of the constructed modules, since intramodular genes cannot be the shared intermodular targets in more than two different modules. In addition, both intermodular Pearson’s R and p-value (one-way ANOVA with Tukey’s multiple comparison) were given for analyzing the correlation between modules. Highly connected gene modules were identified if the p-value was no larger than 0.05.

### Functional Analysis For Gene Modules Across Nasal and Lung Samples

To identify the gene modules characterized by regulating Type I interferon signaling pathway, both gene ontology (GO) and Kyoto Encyclopedia of genes and genomes (KEGG) enrichment analysis were adopted to analyze the biological function of each module. The annotation analysis was resulted by the online database DAVID (https://david.ncifcrf.gov/tools.jsp). When a p-value less than 0.05 was considered significant. Those gene modules with enriched annotation of “Type I interferon pathway” will be collected as the filtered ones for further cross-specimens study. In particular, enriched annotation associated with both “Defense response to virus” and “Type I interferon signaling pathway” would be filtered with UpsetR analysis and for further intensive study. The gene expression atlases (3D view) of nasal and lung specimens would be visualized by R function “plot3D”. Additionally, the key interactive genes functionally representing both “Type I interferon pathway” and “Defense response to virus” were gathered and visualized by Sankey diagram using R function “dplyr” and “networkD3”. Moreover, the QIAGEN Ingenuity Pathway Analysis will be used for mining the pathophysiologic relationship among targets based on reported experimental evidence. The pathway figure was plotted by BioRender and Adobe illustrator (**Supplementary Material 6**).

### Adjusted Rand Index-Based Similarity Analysis For Homogeneous Modules

The Rand index (RI) is an accuracy value for similarity analysis between actual or predicted clusters using the Permutation Model in terms of the following equation: $RI=(\text{a}+\text{b})/\left({}_{2}^{n}\right),$ Where “a” represents the number of genes in the identical gene module, “b” points to those in the distinct modules, while “n” is the entire number of sample groups. However, the premises of the permutation model are violated in certain clustering conditions, such as fixed cluster number with various interpreted data. Thus, the Adjusted Rand Index (ARI) may guarantee the random assignment of variables, which symmetrically measures the similarity and co-expression between assignments. The calculation of Adjusted Rand Index is as follows:


$$\text{ARI}=\frac{2\Sigma_{ij}\left({}_{2}^{nij}\right)-2\left[\Sigma_{i}\left({}_{2}^{ai}\right)\Sigma_{j}\left({}_{2}^{bj}\right)\right]/\left({}_{2}^{n}\right)}{\left[\Sigma_{i}\left({}_{2}^{ai}\right)+\Sigma_{j}\left({}_{2}^{bj}\right)\right]-2\left[\Sigma_{i}\left({}_{2}^{ai}\right)\Sigma_{j}\left({}_{2}^{bj}\right)\right]/\left({}_{2}^{n}\right)}$$

Where n_ij_, a_i_, and b_j_ are values from a contingency matrix with two random sets created by R function “fossil”. The range of ARI varies from −1 to 1, in which negative values represents the independent modules and a positive ARI stands for the similar modules (1 for an approximate perfect match between two modules).

### Transcriptional Factors For Identified Type I Interferon-Related Genes

Transcriptional factor analysis offers sequence upstream of transcriptional start site of interferon-regulated genes, which were colored in the blocks for predicting the potential binding transcription factor in the promoter. The procedure of prediction was based on the MATCH algorithm by TRANSFAC 2012 matrices with minimum false positive cut-off. The transcriptional factor analysis was performed on the Interferome Database V2.0 (www.interferome.org).

## Results

### Constructing Weighted Co-Expression Gene Modules in Nasal Specimen and Pulmonary Tissue Infected by SARS-CoV-2

**Figure 1:**
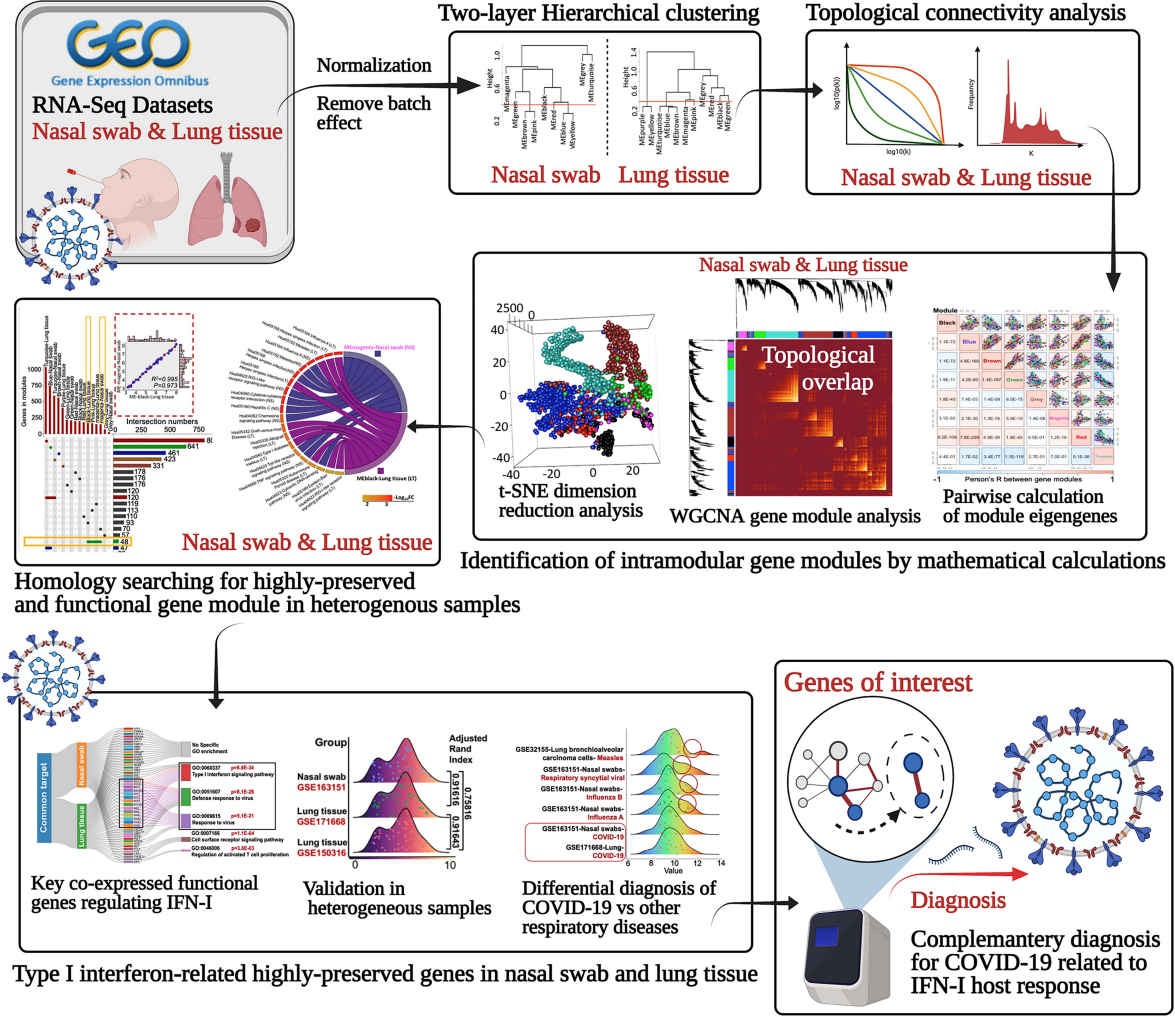
Schematic of identifying the underlying the highly-preserved genes from heterogeneous samples for COVID-19 diagnosis.

Identification of highly correlated consensus gene could disclose the regulatory mechanisms with biologically or pathologically relevant genes that may be potentially mediated. Since WGCNA can establish a scale-free gene network in terms of expression correlation, it can detect interconnected genes and modules characterized as co-expression functionally ones with specific biological profile (28). Therefore, the hub genes and modules may play a dominant role as the representative diagnostic or therapeutic targets for COVID-19 management (**Figure 1**).

**Figure 2:**
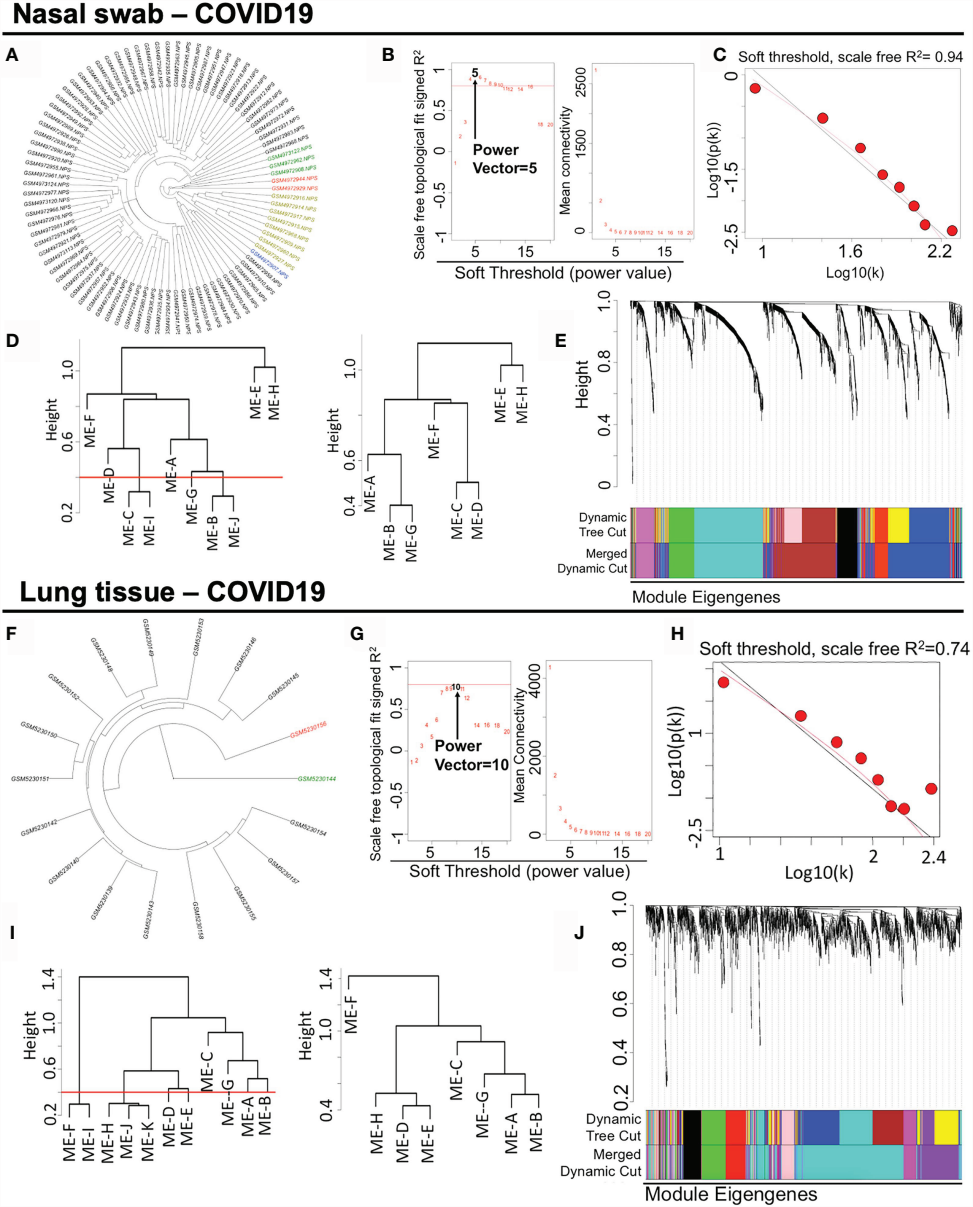
Constructing weighted co-expression gene modules from nasopharyngeal specimen and pulmonary tissue in COVID-19. **(A**, **F)** Hierarchical clustering of genes in nasal swab and lung tissue. The coloring samples are the outliers due to the numerous missing or zero-variance expression genes. **(B**, **G)** Analysis of network topology for various soft-thresholding powers. Left panel shows the index of scale-free topology fit (y-axis) as a function of the soft-thresholding power (x-axis). Right panel shows the value of mean connectivity (y-axis) as a function of soft-thresholding power (x-axis). **(C**, **H)** A log-log plot of connectivity of signed adjacency matrices. The x-axis stands for the logarithm of entire network connectivity, while y-axis is logarithm of related frequency distribution **(D**, **I)** Hierarchical clustering and merging of close-distance gene patterns. **(E**, **J)** A dendrogram composed of relationships among clustered gene modules with high adjacency.

For the datasets acquisition, we adopted the available RNA-sequencing data (*H. sapiens*) with SARS-CoV-2 infection from GSE163151 (nasal swabs) and GSE171668 (lung tissue) in NCBI GEO database. Of note, surviving or postmortem samples with SARS-CoV-2 infection may also unveil the severity of COVID-19 in patients. The nasal swab specimen (GSE163151) may correspond to the early/moderate SARS-CoV-2 infection, while the postmortem pulmonary tissues (GSE171668) probably point to the late infective stage, suggesting the underlying specific gene/module profiles may be demonstrated in a stage-dependent manner across heterogeneous specimens. For data processing, after DESeq2-based data normalization and outlier elimination as described in the method part, we consequently included COVID-19-related nasopharyngeal swabs (n = 73 in GSE163151) and lung autopsy tissues (n = 16 in GSE171668) for WGCNA analysis as shown in black lines in **Figures 2A, F**. The filtered genes (n = 2,649 in nasal swab; n = 2,369 in lung tissue) with expression variance greater than 90% of the whole genome were involved as well. For establishing a scale-free network for clustering gene modules, weighted Pearson’s correlation coefficient β (power value) was selected in accordance with relatively high value of signed R^2^ resulted from scale-free topological analysis. Therefore, β = 5 (nasal swab) and β = 10 (lung tissue) were used for constructing the scale-free clustering dendrograms (**Figures 2B, G** **)**. The signed R^2^ was shown in a log-log linear model for module connectivity analysis is R^2^ = 0.94 (nasal swab) and R^2^ = 0.74 (lung tissue), along with the mean connectivity close to 0 for both specimens, suggesting the successful construction of scale-free correlation for WGCNA analysis (**Figures 2C, H** **)**. Thus, after hierarchical clustering and merging the close-distance gene patterns, eight gene modules for both nasopharyngeal specimens and lung tissues were identified (**Figures 2D, I** **)**. More specifically, eight gene modules in nasopharyngeal dataset were as follows: ME-A (191 genes), ME (module)-B (741 genes), ME-C (558 genes), ME-D (208 genes), ME-E (23 genes), ME-F (145 genes), ME-G (194 genes), and ME-H (589 genes), while eight gene modules in postmortem lung tissue were shown as below: ME-A (165 genes), ME-B (215 genes), ME-C (67 genes), ME-D (147 genes), ME-E (161 genes), ME-F (403 genes), ME-G (177 genes), and ME-H (1034 genes). The relationships among clustered gene modules with high adjacency were visualized in the dendrograms (**Figures 2E, J** **)**. All the supporting data for gene module classification were shown in the **Supplementary Materials 1**, **2**. Herein, weighted co-expression gene modules in SARS-CoV-2 infected nasal specimen and pulmonary tissue were established for further analysis.

### Impact of SARS-CoV-2 Infection on the Intramodular Correlation of Co-Expression Gene Modules in Nasal Swabs and Lung Tissues

**Figure 3:**
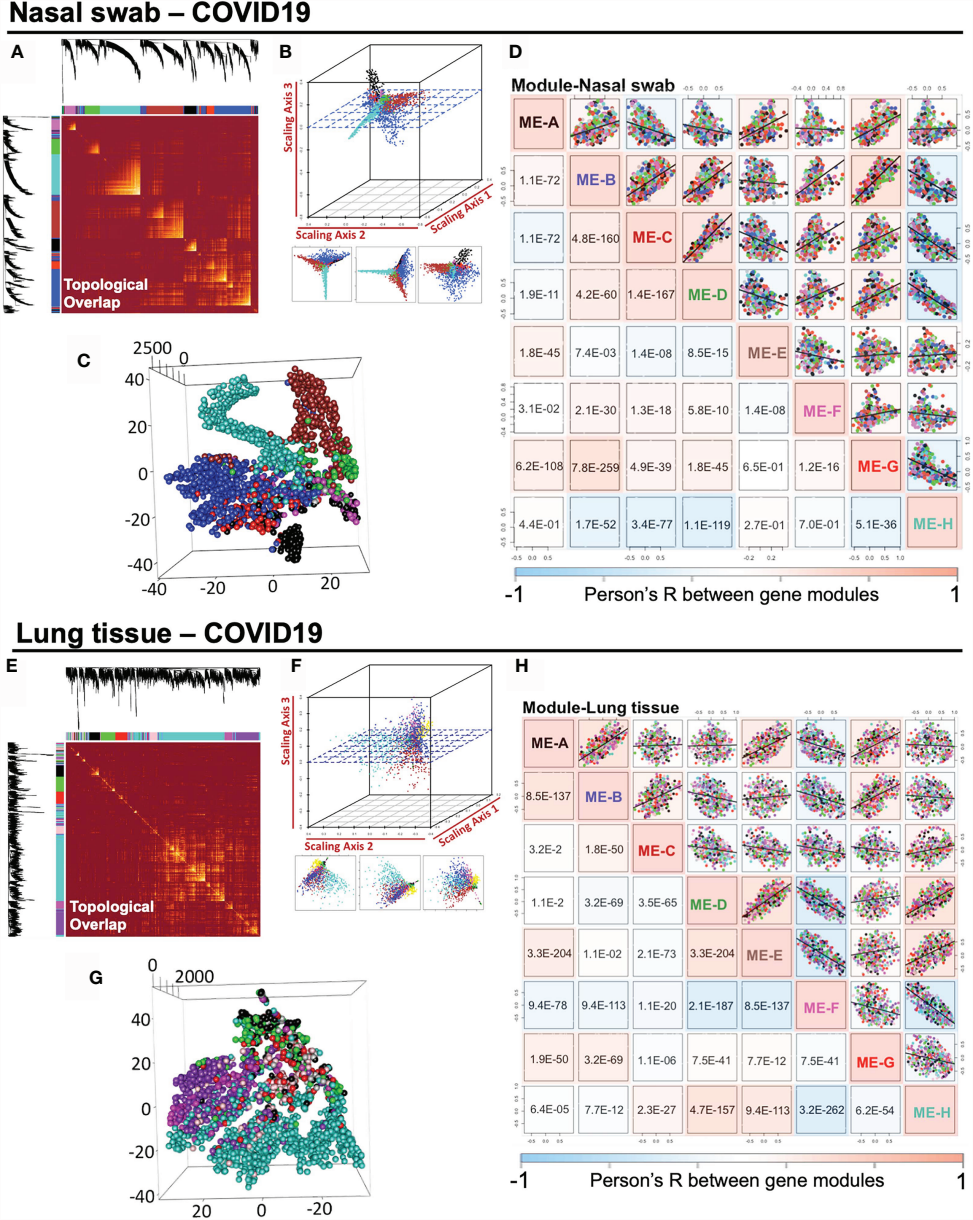
Impact of SARS-CoV-2 infection on the intramodular correlation of co-expression gene modules in nasal swabs and lung tissues. **(A**, **E)** A pairwise association of gene modules in a topological overlap plot. **(B**, **F)** Geometric data analysis in 3D scattering. **(C**, **G)** t-SNE dimension reduction analysis to observe the distribution of gene modules. **(D**, **H)** Pairwise illustration of gene module correlation profile indicated by Pearson’s R shown in whole pairwise scatterplots in color with p-value in the lower panel (one-way ANOVA with Tukey’s multiple comparison).

COVID-19-induced host response in nasal and lung tissues may share common regulated pathway with antiviral effects and innate immunity. Highly correlated co-expression gene modules may be pivotal in mediating pathological actions. Thus, we measured the module relationship in accordance with their Module Eigengene (ME) values as described in *Methods* section. To begin with, it was straightforward to illustrate the pairwise association of gene modules in a topological overlap plot (**Figures 3A, E** **)**. Co-expressed gene modules were colored with yellow, which are widely shown in the topological plot. Notably, intramodular genes cannot lie “intermediate” across distinct modules, which would fail to be strong connected intramodular targets in either module. Taken advantage of geometric data analysis in multiple dimensions, we adopted the 3D scattering approach (**Figures 3B, C** **)** and t-SNE dimension reduction analysis (**Figures 3F, G** **)** to observe the distribution of gene modules, indicating that the intramodular genes were potentially spread separately. It was demonstrated that there was no gene intersection across modules, suggesting the qualified composition of heterogeneous gene modules (**Supplementary Materials 1**, **2**). Also, the intramodular genes may be potentially dedicated together to a specific pathway. For examples, ME-H-Lung is related to “GO:0016032 viral process (p = 4.95E−07) and “hsa04330: Notch signaling pathway (p = 3.20E−04)”, while ME-H-Nasal swab points to “GO: 0003341 cilium movement (p = 4.41E−17)” and “hsa05016: Huntington’s disease (p = 4.90E−06)”. Besides, ME-B-Nasal swab stands for “GO:0001569 patterning blood vessels (p = 1.52E−04)” and “hsa04974: Protein digestion and absorption (p = 1.51E−3)”. Before conducting detailed functional analysis, we would like to initially measure the correlation profile among gene modules, which was further analyzed by ME-dependent Pearson’s R shown in whole pairwise scatterplots in color with a p-value in the lower panel (one-way ANOVA with Tukey’s multiple comparisons) (**Figures 3D, H** **)**. Surprisingly, in 28 times linear regression calculation among eight modules in either nasal or lung specimens, the pairwise p-value lower than 0.05 were 24/28 for nasal swab and 28/28 for lung tissue, indicating the strong co-expression correlations among modules in the same sample. Taken together, our findings in this part were concluded as follows: 1) The scale-free network of consensus modules may be well-established without the noise of cross-module genes. 2) Activation of a specific functional module may result in a cascaded fluctuation of certain genomic functions regulated by remaining dependent modules. 3) As a molecular strategy against COVID-19, screening out a highly-preserved functional module in a scale-free network from heterogeneous samples may be meaningful for COVID-19 diagnosis.

### High Preservation of Type I Interferon Pathway Specific Gene Modules From Nasal Swab and Postmortem Lung Tissue Infected With SARS-CoV-2

**Figure 4:**
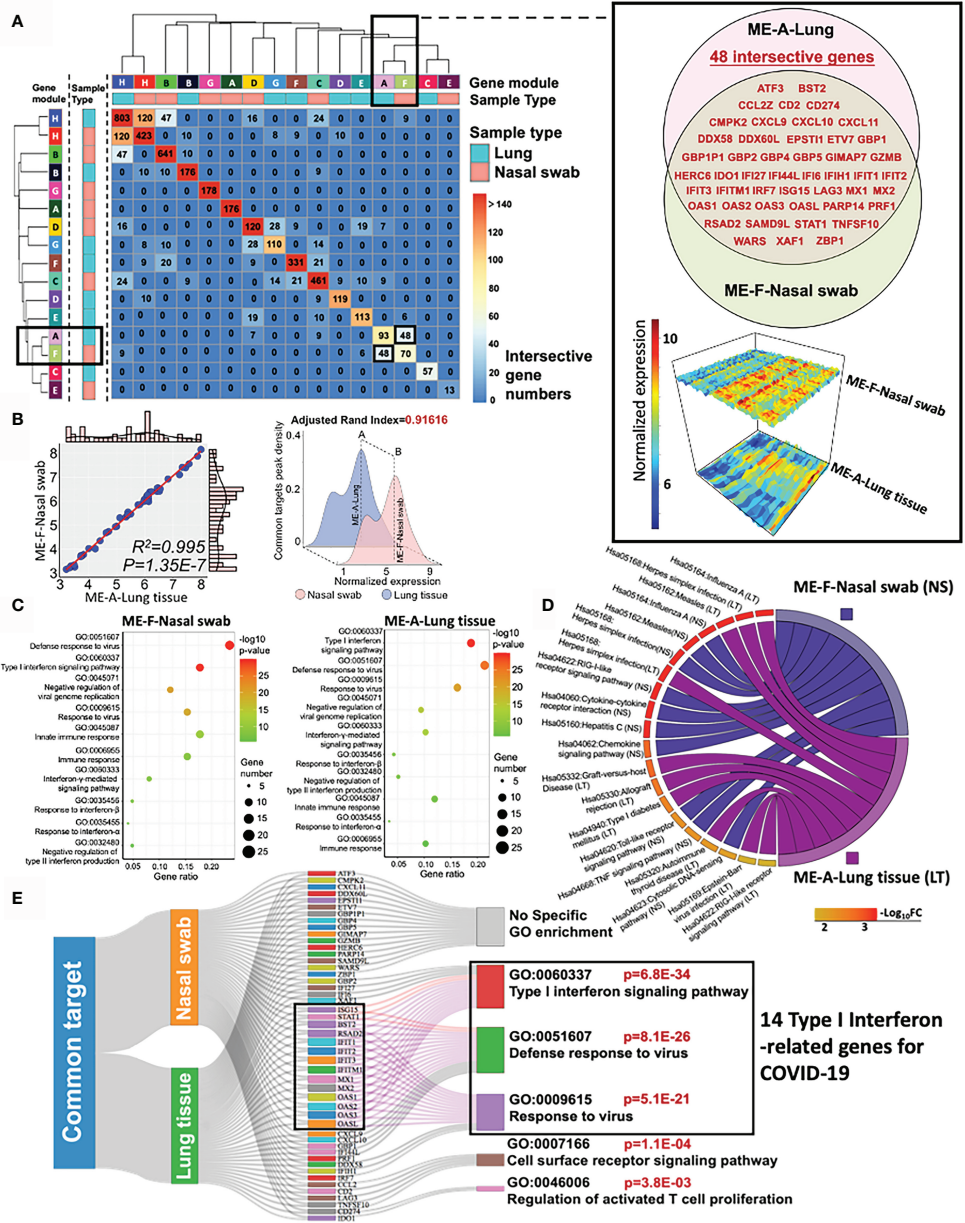
High preservation of Type I interferon gene from nasal swab and postmortem lung tissue in COVID-19. **(A)** A two-dimensional matrix shows the overlap genes in modules from nasal swab or lung tissue. The black rectangles represent the interesting gene module showing a large proportion of overlapped genes in either ME-A-Lung or ME-F-Nasal swab. The detailed information of intersective genes with normalized expressions from ME-F-Nasal swab and ME-A-Lung are shown in the right panel. **(B)** Linear relationship and adjusted rand index between ME-F (Nasal swab) and ME-A (Lung). **(C, D)** GO and KEGG functional enrichment analysis for ME-F (nasal swab) and ME-A (lung tissue). **(E)** Functional genes for corresponding enrichment annotations shown in a Sankey diagram.

To evaluate the similarity of specific co-expression gene modules in nasal and lung specimens, we measured the intersective genes, expression profile, biofunction, and reproducibility (adjusted rand index) of modules across distinctive samples by R function “Upset R”, “pheatmap”,”plot3D”, and “fossil”, respectively. A two-dimensional matrix was plotted for showing the overlap genes in modules from nasal swab or lung tissue (**Figure 4A**). It indicated that “ME-H-Lung and ME-H-Nasal swab” (120 genes), “ME-A-Lung and ME-F-Nasal swab” (48 genes), and “ME-H-Lung and ME-B-Nasal swab” (47 genes) have the most intersective genes (Top 3). However, only approximately one-ninth genes are overlapped in ME-H-Lung with either ME-H-Nasal swab or ME-A-Lung, suggesting the limited functional contribution of these intersection genes with “ME-H-Lung”. Furthermore, the functional overlap is absent among these three modules as shown in the functional analysis of above paragraph. On the other hand, the overlapped genes (n = 48) in “ME-A-Lung and ME-F-Nasal swab” (also shown in the Venn diagram) has a large proportion in ME-F-Nasal swab (48/145, GSE163151) and ME-A-Lung (48/165, GSE171668). showing that approximately one-third of the whole genes are the overlapped genes in either ME-F-Nasal swab or ME-A-Lung. The gene names and normalized expression (gene atlas) were shown in the right panel of **Figure 4A**. For measuring the correlation between ME-F-Nasal swab or ME-A-Lung, linear regression and adjusted rand index analysis were performed. As a result, mean expression-based Pearson’s R^2^ (R^2^ = 0.995) and p-value (p = 1.35E−7) indicated a significant correlation and similarity between these two modules from heterogeneous samples (**Figure 4B**, left panel). The highly-preserved gene profile is further validated by adjusted rand index-dependent homogeneous analysis (adjusted rand index = 0.91616) (**Figure 4B**, right panel). R function “SVA” was used to eliminate the batch effects of normalized gene expression in both datasets prior to conducting adjusted rand index analysis. Both outcomes suggested the high preservation between ME-F-Nasal swab and ME-A-Lung. Prompted by these findings, both GO and KEGG analyses were adopted for functional enrichment analysis (**Figures 4C, D** **)**. Strikingly, host responses for COVID-19 in ME-F-Nasal swab are as follows:” GO:0051607 Defense response to virus (p = 9.72E−31)”, “GO: 0060337 Type I interferon signaling pathway (p = 7.58E−30)”, and “Hsa05164: Influenza A (p = 7.36E−08)”. For lung tissue, the enriched pathways in ME-A-Lung are “GO: 0060337 Type I interferon signaling pathway (p = 2.01E−30)”, “GO:0051607 Defense response to virus (p = 2.32E−25)”, and “Hsa05164: Influenza A (p = 9.29E−11)” suggesting the consistent functional gene modules between ME-F-Nasal swab and ME-A-Lung. Of note, both host responses “Type I interferon signaling pathway” and “Defense response to virus” were predicted as the two common regulated pathways detected in SARS-CoV-2 infected nasal swab and lung tissue, suggesting the high functional preservation of Type I interferon-related genes between nasal and lung tissues in response to COVID-19. Here was the summary of this section: 1) SARS-CoV-2-stimulated “Type I interferon pathway” was an underlying highly-preserved signaling in nasal and lung samples. 2) It may be beneficial for COVID-19 diagnosis and therapy by a deep understanding of COVID-19-induced fluctuation of key co-expression genes related to both “Type I interferon pathway” and “Defense response to virus” in highly preserved modules (ME-F-Nasal swab and ME-A-Lung) across heterogeneous samples. Herein, GO/KEGG enrichment with Sankey diagram analysis was adopted to functionally select the concordant genes associated with “Type I Interferon signaling pathway” and at least one of the two annotations, including “Defense response to virus” and “Response to virus” (**Figure 4E**). As a result, 14 common genes were identified as the preserved co-expression ones in nasal swab and lung tissue for mediating Type I interferon signaling in COVID-19. The 14 of 48 genes were as follows: BST2, IFIT1, IFIT2, IFIT3, IFITM1, ISG15, MX1, MX2, OAS1, OAS2, OAS3, OASL, RSAD2, and STAT1, suggesting the potential diagnostic role for COVID-19. Therefore, the bioinformatic validation shows the high preservation of 14 IFN-I-related genes across nasal swab and lung tissue, showing a concordant diagnostic biosignature for COVID-19 diagnosis.

### Stratified Diagnose of COVID-19 Severity in Terms of the 14 Type-I Interferon-Inducible Genes

**Figure 5:**
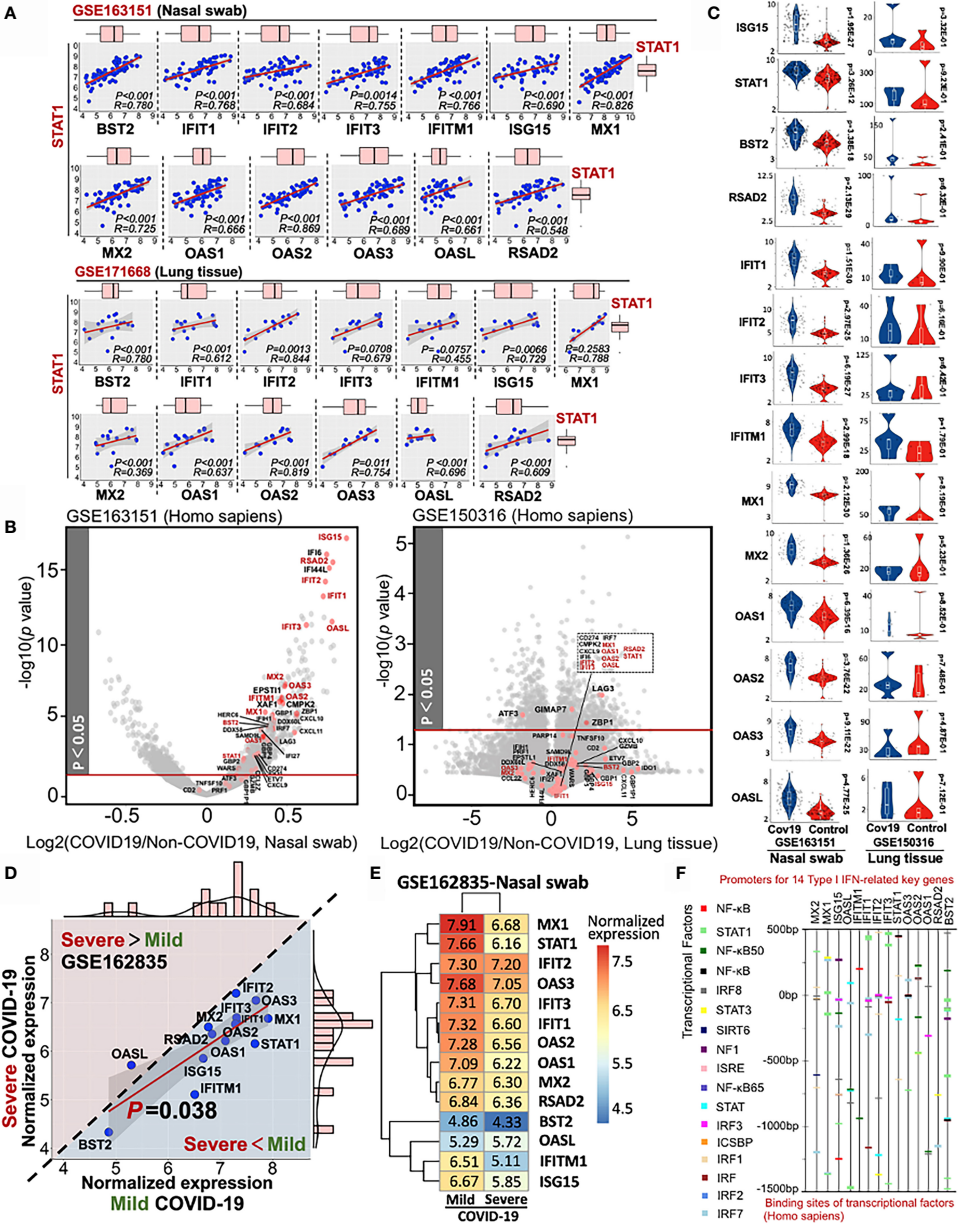
Stratified diagnose of COVID-19 severity in term s of 14 Type-I interferon-inducible genes. **(A)** Linear relationship between STAT1 and other 13 IFN-related genes. **(B)** Volcano plots of differential gene profile in GSE163151 (nasal swab) and GSE150316 (lung tissue) (COVID-19 vs non-COVID-19). Red points shown in the volcano plots stand for the intersective genes across nasal swab and lung tissue. **(C)** Differential transcriptional expression of 14 IFN-I-related genes with or without SARS-CoV-2 infection **(D)** Comparative analysis of the 14-gene transcriptional profile (GSE162835) between mild and severe COVID-19. A diagonal line divides two different expression trends. The upper panel (pink background) indicates the positive correlation between disease severity and gene expression, while the negative correlation (blue background) is shown in the lower panel. **(E)** A heatmap showing the values of normalized expression of 14 genes in either mild or severe COVID-19. **(F)** Potential transcriptional factors binding to the promoter sits of 14 key genes analyzed by Interferome database.

Using QIAGEN Ingenuity Pathway Analysis, we further identified the pathophysiologic correlation among these 14 IFN-I-related genes. All the target connections are based on the reported experimental evidence, showing that transcriptional factor STAT1 may play a central role in the 14-gene network for COVID-19 diagnosis (**Supplementary Material 6**). Moreover, the multiple linear regression results indicated the strong linkage among STAT1 and other 13 IFN-I-related genes in heterogeneous samples (**Figure 5A**). All the p-value of pairwise analysis (STAT1 vs other 13 genes) in nasal swab was lower than 0.05 (13/13), while the p-value for most of the STAT1-dependent paired comparison in lung tissue was less than 0.05 (10/13). Although 14 IFN-I-related genes as host response to COVID-19 were potentially preserved across nasal and lung specimens, the differential transcriptional expression of genes in samples with or without SARS-CoV-2 infection remains obscure. Understanding the differential profile of genes is conductive to realize the relationship between vulnerability of interferon activity and COVID-19 severity. since GSE171668 (lung tissue) was absent of negative control for COVID-19 study, we analyzed the differential expression of 48 intersective genes in GSE163151 (nasal swab) and GSE150316 (lung tissue). As shown in the volcano plots (COVID-19 vs non-COVID-19 in homogeneous samples), 48 intersective genes were highlighted in red points, in which gene names in red pointed to the 14 genes regulating IFN-I pathway. Interestingly, the volcano plot indicated a significant increase of 48 intersective genes expressions (including 14 IFN-I genes) in nasal swab (COVID-19 vs non-COVID-19), but a decreased profile in postmortem lung tissue (**Figure 5B**). More specifically, the transcriptional changes of 14 IFN-I-related gene with or without COVID19 infection were further shown in **Figure 5C**, indicating that SARS-CoV-2 infection induced a robust IFN-I response in nasal swab (14/14, p <0.05), but a decreased response in postmortem lung tissue (0/14, p <0.05). The nasal swab specimen (GSE163151) may relate to the early/moderate SARS-CoV-2 infection, while the postmortem pulmonary tissues (GSE171668) probably point to the late infective stage. For further addressing this issue, GSE162835 was used to measure the “gene expression-severity” relationship of these 14 genes (**Figure 5D**). GSE162835 contains transcriptional data with disease severity. As a result, a 14-gene based linear relationship was detected between mild and severe COVID-19 (p = 0.038). Additionally, the expressions of genes (13/14, except OASL) were negatively correlated with COVID-19 severity (areas below the diagonal line in **Figure 5D**). The normalized expressions of 14 genes in the nasal swab of mild or severe COVID-19 were further shown in a heatmap (**Figure 5E**). Consistent with our result, scientists have reported that the increasing level of IFN-I-related genes in the onset of COVID-19 is reversed in the late stage due to the enhanced load of SARS-CoV-2 virus (29). Taken together, the 14-gene profile may be responsible for stratifying the COVID-19 severity. Additionally, for in-depth understanding the regulatory role of these 14 genes, we further investigated the potential transcriptional factors capable of binding to the promoters of these 14 key genes using Interferome database (30). The predicted transcriptional factors mainly consisted of NF-κB, STAT, and IRF family (**Figure 5F**). In sum, based on RT-PCR virus detection, the highly-preserved 14 IFN-I-related genes between nasal swab and pulmonary tissue may further complement diagnosis of COVID19 with its severity.

### Differential Diagnosis of COVID-19 With Other Respiratory Diseases by a 14-Gene Expression Profile

**Figure 6:**
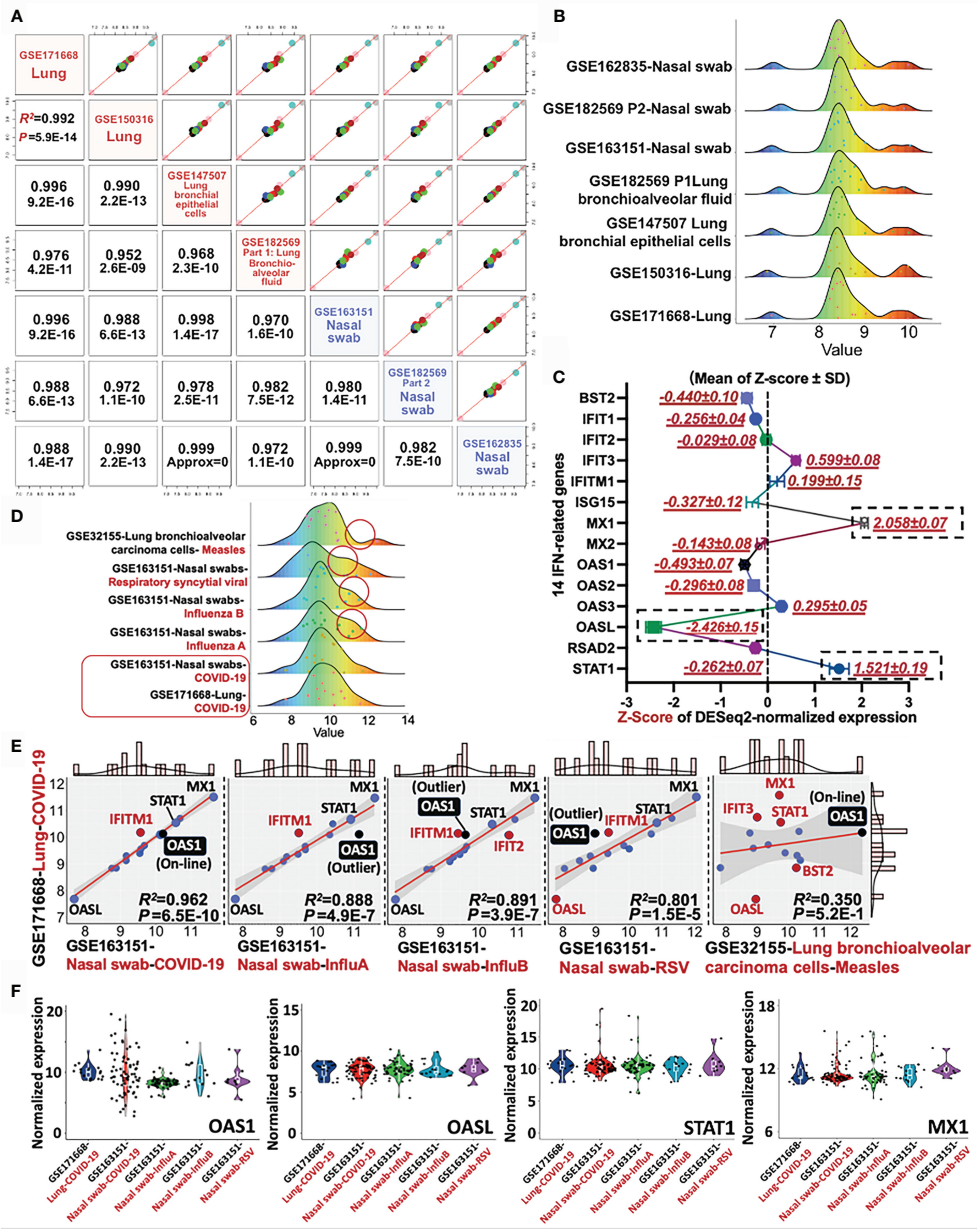
Differential diagnosis of COVID-19 with other respiratory infectious diseases by a 14-gene expression profile. **(A)** Pairwise analysis of gene profile in heterogeneous samples in COVID-19, including four nasal swabs (GSE163151, GSE162835, and GSE162835); two lung tissues (GSE171668 and GSE150316), and one lung bronchial epithelial cells (GSE147507). Pearson’s correlation coefficient R and P-value are shown in red. **(B)** Trendgram of normalized 14-gene expression in heterogeneous samples infected by SARS-CoV-2. **(C)** Representative trendgram from SARS-CoV-2 infected samples. It is quantitatively mapped by Z-score normalization of gene expression from databases shown in panel **(A)**. The three rectangles represent the absolute value of Z-score with large variations from the baseline (Top 3). **(D)** Representative Trendgram of COVID-19 and other respiratory diseases (Influenza A, Influenza B, RSV, and Measles). Red circles indicate the distinct peak trend when compared with that of COVID-19. **(E)** The linear relationship between COVID-19 and other respiratory infections, in which the outlier genes are shown in red. In particular, the point of OAS1 is highlighted in black. The value of Pearson’s R-square and P value between datasets are indicated in the right bottom panel. **(F)** Violin plots shown the normalized expression of OAS1, OASL, STAT1, and MX1 from various datasets.

Since the 14 IFN-I related genes are classical STAT-IRF-associated genes, which can be triggered by other viruses rather than SARS-CoV-2 alone, it is essential to conduct a differential diagnosis between COVID-19 and other respiratory infectious diseases. To address this issue, we further included the RNA-sequencing data of Influenza A, Influenza B, Respiratory syncytial viral (RSV), and Measles (GES32155, GSE163151, and GSE171668) for the differential analysis. Firstly, the consistence of 14-gene profile was determined in heterogeneous COVID-19 samples. Apart from previous used datasets (GSE171668-lung tissue and GSE150316-lung tissue), additional four datasets were further included for pairwise analysis. The other COVID-19-related four datasets include GSE147507 (lung bronchial epithelial cells), GSE163151 (nasal swab), GSE162835 (nasal swab), and GSE182569 (lung bronchioalveolar fluid and nasal swab). As a result, the minimum and maximum value for Pearson’s R-square and p-value for all paired tests are R^2^ = 0.952 and p = 2.6E−9 respectively, suggesting the highly-conserved transcriptional profile of the 14 IFN-related genes in heterogeneous samples (nasal swab, lung tissue, bronchioalveolar fluid) (**Figures 6A, B** **)**. For the establishment of a molecular reference for COVID-19 diagnosis, we quantitatively mapped a trendgram by Z-score quantification of 14-gene expression for COVID-19 specific diagnosis. In **Figure 6C**, the Z-score values of MX1 (2.058 ± 0.07), OASL (−2.426 ± 0.15), and STAT1 (1.521 ± 0.19) shown in black rectangles exhibit large variance from the baseline (Top 3). The data resource for plotting 14-gene Z-score trendgram was retrieved from COVID-19 datasets as shown in **Figure 6A**. Besides,trendgrams of 14-gene normalized expression in various diseases, including COVID-19, Measles, Respiratory Syncytial Viral (RSV), and Influenza A/B, were plotted for detecting the expression difference. Red circles indicate the distinct peak trend when compared with that of COVID-19, suggesting the existence of expression distinction between COVID-19 and other infections **Figure 6D**. For deepen understanding of differential diagnosis, linear regression analysis was used for further distinguishing the type of viral infection. In **Figure 6E**, firstly, the genetic profile of 14 genes in Measles is linearly irrelevant with that of COVID-19 (R^2^ = 0.35, Pearson’s p = 0.52), suggesting the feasibility of 14-gene based differential diagnosis between COVID-19 and Measles. For COVID-19 in comparison with other respiratory infections (Influenza A/B or RSV), it was resulted that the R^2^ between COVID-19 samples is higher (R^2^ >0.9) than that of COVID-19 vs Measles/RSV/Influenza A/B (all R^2^ <0.9). Besides, the Pearson’s p-value for COVID-19 vs COVID-19 samples (p = 6.5E−10) is at least 1,000 times lower than that of COVID-19 vs Influenza A/B and RSV (all p ≤4.9E−7), suggesting the high-preservation of 14 genes among COVID-19 samples. However, although the Pearson’s correlation of “COVID-19 vs COVID-19” is much stronger than that of “COVID-19 vs Influenza A/B or RSV”, the correlative level is still significant in “COVID-19 vs Influenza A/B or RSV” (P <0.05). It is therefore essential to discover a diagnostic host classifier that can distinguish among COVID-19 and Influenza A/B/RSV. Notably, although the Z-scores of OASL, STAT1 and MX1 were far away from the Z-score baseline within a 14-gene profile, their relative expression (locations) in linear models are approximately similar, showing the invalid potential for differential diagnosis (**Figure 6E**). However, among the IFN-I related 14 genes, OAS1 may play a complementary role in the differential diagnosis of COVID-19 and Influenza A/B/RSV. The reason is that the expression value (point location) of OAS1 is on the linear regression line in the comparison of “COVID-19 vs COVID-19” in heterogeneous samples, but it is an outlier of the regression line and 95% confidence interval in comparison of “COVID-19 vs InfluenzaA/B/RSV” (**Figure 6E**), indicating an underlying diagnostic classifier of OAS1 in a 14-gene regression model. The normalized expression of representative genes, including OAS1, OASL, STAT1, and MX1, were shown in the violin plots **Figure 6F**, which aims to visualize the transcriptional expression of characteristic genes from 14-gene regression models and a Z-score trendgram. In sum, it is prospected that based on the direct determination of SARS-CoV-2 virus by RT-PCR, the detection of transcriptional profile of these 14 IFN-I related genes may be a promising molecular reference for COVID-19 diagnosis.

## Discussion

Accumulating evidence has indicated that direct testing of SARS-CoV-2 virus may cause false-negative results by RT-PCR due to unstable viral loads and evolution. Besides, genetic profiles of host defense have been demonstrated to be able to recognize the specific bacterial or viral infection (31). Thus, discovering the unique transcriptional feature of COVID-19 may supplement the diagnostic strategy of COVID-19, especially the signature of host response. Notably, SARS-CoV-2-induced changes of gene expressions has reported to potentially distinguish COVID-19 from other infections (e.g., MERS-CoV and SARS-CoV), in which IFN-I genes is involved in the unique biosignature in response to SARS-CoV-2 infection (32). However, ISGs can be triggered by various stimulators. Thus, even used as a supplementary molecular reference, the diagnostic signature of specific ISGs should be representative for COVID-19. In this study, using transcriptional data RNA-Seq from GEO datasets, we identified highly-preserved genes/modules regulating IFN-I pathway in SARS-CoV-2-infected nasal swab and lung tissue by R-dependent machine-learning analysis. It intends to provide a complementary understanding of IFN-I-related host response as a diagnostic indicator of COVID-19 and its severity.

For explicitly recapitulating our findings, firstly, we constructed a scale-free co-expression gene network without any preliminary assumption in terms of the normalized transcriptional data (RNA-seq datasets from GEO database) by WGCNA, since the real-world biological relevance commonly represents scale-free behaviors. After establishing the gene modules shown in the gene clustering dendrograms from either nasal or lung samples, the scale-free network was well-established with the absence of cross-module genes. Almost all the constructed pairwise modules in nasal swab (24/28) and lung tissue (28/28) were significantly correlated, indicating that screening out a predominant functional module may play a representative role in a genomic network. In homology research, highly-preserved genes/modules in heterogeneous specimens usually possess significant biological functions with similar drivers or regulators. It may be meaningful for demonstrating susceptible gene targets in an identical disorder. Using adjusted rand index-related similarity analysis and UpsetR, we identified a highly-preserved genetic profile (n = 48) across the nasal swab and lung samples infected by SARS-CoV-2. Coincidently, biological function of the 48 intersective genes pointed to both “Type I interferon signaling pathway” and “Defense response to virus”, suggesting that “regulating IFN-I-related signaling pathway against virus” may be highly conserved in nasal swab and lung tissue. Based on these findings, we identified 14 IFN-I-related genes as the most dominant functional genes among these 48 genes, which were resulted from highly-enriched annotations including “Type I Interferon signaling pathway” and at least one of the two annotations, including “Defense response to virus” and “Response to virus”. The highly preserved IFN-I related 14 genes are as follows: BST2, IFIT1, IFIT2, IFIT3, IFITM1, ISG15, MX1, MX2, OAS1, OAS2, OAS3, OASL, RSAD2, and STAT1. These 14 genes are also documented to involve in the host response to COVID-19 (29). The potential interaction between these 14 genes and virus have been reported as follows: BST2 was found to inhibit viral egress antagonized by SARS-CoV-2 accessory protein Orf7a (33); IFIT1/2/3 may inhibit the translation and replication of virus (34); IFITM1 possibly block membrane fusion of diverse enveloped virus (35); ISG15 conjugated with virus is vital for IFN-related host antiviral responses regulated by the viral RNA sensors, including MDA5 (36); MX1 has a potential suppressive effect on the activity of viral ribonucleoprotein complex and its GTPase (37). MX2 may be effective in repressing viral replication, transcription, and nucleocapsid shuttling (38); OAS1/2/3 potentially focused on inhibiting viral replication, while OASL is associated with viral translation (39). RSAD2 has underlying anti-viral egress and replication effect (40). IFN-related STAT1 nuclear translocation is the indispensable process for antiviral transduction (41). Additionally, SARS-CoV-2 is documented to suppress interferon and STAT activity, resulting in the clinical manifestation of COVID-19. In our findings, STAT may be the central role of this 14-gene network for COVID-19 diagnosis (**Supplementary Material 6**).

On the other hand, using seven independent RNA-sequencing datasets from NCBI-GEO, we further mathematically validated the high preservation of this 14-gene profile in homogeneous and heterogeneous samples infected by SARS-CoV-2, supporting the diagnostic role of 14-gene profile for COVID-19. Besides, consistent with previous results, based on GSE162835 (RNA sequencing data with labeled COVID-19 severity), our study suggested that sufficient expression of these14 IFN-I genes is in an early stage of COVID-19, while it is decreased in a severe period (**Figure 5D**). Moreover, it is further reported in a clinical trial that the expression of 8/14 genes: BST2, MX1, OAS1, IFIT1, IFITM1, ISG15, RSAD2, and STAT1, were relatively decreased in patients with advanced stage of COVID-19 when compared with that in the early stage, which further evidence the diagnostic feature of this 14-gene profile for COVID-19 severity (42). Apart from these findings, we reviewed the transcriptional factors binding to 14 IFN-I gene-related promoter sites in the Interferome Database, showing that a series of NF-κB, STAT, and IRF family members have potential regulatory capability for these 14-IFN genes. For biological interpretation, the activity of NF-κB is a double-edged sword. Normal binding between NF-κB and IFN is functioned by regulating cell survival and innate/adaptive immune responses, while aberrant NF-κB may contribute to inflammation (43). IFN activity is linked with JAK/STAT-dependent innate antimicrobial immunity (44). All types of IFNs are able to produce STAT by JAK-induced tyrosine phosphorylation (45). STAT1 mutations can result in IFN-related infections and inflammation (46). Nevertheless, IFN-dependent anti-pathogen effect is potentially associated with the absence of STAT1 (47). STAT1 and STAT2 are both regarded as the primordial signal regulators of IFN-I as functioned by genetic ablation, hypomorphic mutation or abnormal function of impaired antiviral IFN-related genes (48). IRF9 form ISGF3 complex can transactivate IFN-related genes for antiviral response as well (49). STAT3 can be activated by IFN-I stimulation in numerous cell types (50). An anti-inflammation role has been reported in IFN and Toll-like receptor response. Both IRF3 and IRF7 serve as a critical role in IFN-I for combating viral infection if adequate IRF3 and IRF7 bind to the promoter sites of IFN-I genes. IRF3 degradation is highly related with the repression of IFN-β (51). ISG15 expression is highly related with IFN stimulation. Overexpression of ISG15 can accelerate DNA replication fork progression followed by abundant DNA damage and chromosomal breakage (52). Apart from these findings, we further provided additional information for differential diagnosis between COVID-19 and other respiratory infections such as COVID-19 vs Influenza A/B, RSV, and Measles. As the results, the distinction of COVID-19 and Measles can be performed according to the IFN-I related 14-gene expression. Moreover, the expression value (point location) of OAS1 in a 14-gene linear regression model can be used as a diagnostic classifier between COVID-19 and Influenza A/B/RSV, since the expression of OAS1 is on the regression line in the comparison of “COVID-19 vs COVID-19” in heterogeneous samples, but it is an outlier of the regression line and 95% confidence interval in comparison of “COVID-19 vs InfluenzaA/B/RSV. Taken together, these biological and statistical interpretations for the results may further suggest a potential molecular strategy for COVID-19 diagnosis in terms of IFN-I associated 14-gene profile.

For the current COVID-19 diagnosis, the most definitive and accurate approach for measuring genetic profile and virus may be the high-throughput sequencing. However, this method is relatively disadvantage to large-scale application due to the expensive equipment and skillsets required. Moreover, identification of too many differential expression genes may not be representative and precisive enough to COVID-19 diagnosis. Thus, the 14-gene-based transcriptional profile may significantly cut down on manpower and equipment expenditure to the diagnosis. On the other hand, as a sensitive and precise approach widely used in hospitals and laboratories, RT-PCR remains the gold standard for COVID-19 diagnosis (53). Since February 2020, the US Food and Drug Administration (FDA) approved licensed laboratory to detect SARS-CoV-2 virus (20). The procedure includes the isolation and conversion of virus RNA to cDNA followed by the amplification of cDNA using Taq DNA polymerase. Afterwards, RT-PCR using primers will quantitively detect genome parts of SARS-CoV-2 virus. Such a procedure can be used for detecting transcriptional level of interesting genes as well. Notably, in asymptomatic COVID-19 patients, 38% of whom are PCR negative for virus detection (54, 55), suggesting the urgent need for supplementary diagnosis by other biological reference. Because RT-PCR is still effective and sensitive to monitor the transcriptional alterations of IFN-I related genes in asymptomatic COVID-19 patients (23), it indicates the clinical significance of detecting these gene candidates by RT-PCR for supplementary diagnosis. In this paper, for a strict view, we recommended a diagnostic strategy of COVID-19 by simultaneously detecting the genetic profile of both 14 IFN-I related genes (**Figure 6C**) and SARS-CoV-2 viral load. Meanwhile, the severity of COVID-19 is inversely proportional to the transcriptional level of these IFN-I related 14 genes. Taken together, our study may provide a molecular reference for COVID-19 supplementary diagnosis, which is consisted of 14 highly-preserved genes regulating IFN-I-dependent host response in heterogeneous specimens, including nasal swab and lung tissues.

## Conclusion

As noted above, the main merits of this study are as follows: 1) Using transcriptional and computational analysis on RNA-sequencing data retrieved from NCBI-GEO, we identified 14 highly-preserved genes in nasal swab and lung tissues regulating IFN-I-dependent host response for COVID-19. The 14 genes include BST2, IFIT1, IFIT2, IFIT3, IFITM1, ISG15, MX1, MX2, OAS1, OAS2, OAS3, OASL, RSAD2, and STAT1, which may be leveraged for COVID-19 diagnosis with direct virus detection by RT-PCR. The highly-conserved genetic profiles of these 14 genes were validated in SARS-CoV-2-infected homogeneous and heterogeneous specimens, including bronchioalveolar fluid and bronchial epithelial cells. 2) The stratified severity of COVID-19 may also be identified by the transcriptional level of these 14 IFN-I genes. Sufficient transcriptional expression of these 14 genes were peaked in early stage while insufficient in the advanced stage of COVID-19. However, for the limitation of this study, more evidence from clinical trials should be provided for further supporting the 14 gene-based transcriptional profile for COVID-19 supplementary diagnosis

## Data Availability Statement

The datasets presented in this study can be found in online repositories. The names of the repository/repositories and accession number(s) can be found in the article/**Supplementary Material**.

## Author Contributions

YF and NW conceived and designed the study. CZ collected the data. CZ, NW, and YF analyzed the data and drafted the manuscript. Y-GF and CT discussed and revised the manuscript. All authors contributed to the article and approved the submitted version.

## Funding

This study was supported by Hong Kong Chinese Medicine Development Fund (Project Code: 19SB2/002A), Wong’s donation (project code: 200006276), and a donation from the Gaia Family Trust of New Zealand (project code: 200007008).

## Conflict of Interest

The authors declare that the research was conducted in the absence of any commercial or financial relationships that could be construed as a potential conflict of interest.

## Publisher’s Note

All claims expressed in this article are solely those of the authors and do not necessarily represent those of their affiliated organizations, or those of the publisher, the editors and the reviewers. Any product that may be evaluated in this article, or claim that may be made by its manufacturer, is not guaranteed or endorsed by the publisher.
