# Supplementary material for: Transcriptional Profiling and Machine Learning Unveil a Concordant Biosignature of Type I Interferon-Inducible Host Response Across Nasal Swab and Pulmonary Tissue for COVID-19 Diagnosis
Source: Front Immunol. 2021 Nov 22;12:733171. doi: 10.3389/fimmu.2021.733171 (PMC8647662; doi:10.3389/fimmu.2021.733171)
Supplement: Supplementary file 6 [file DataSheet_6.docx]

**
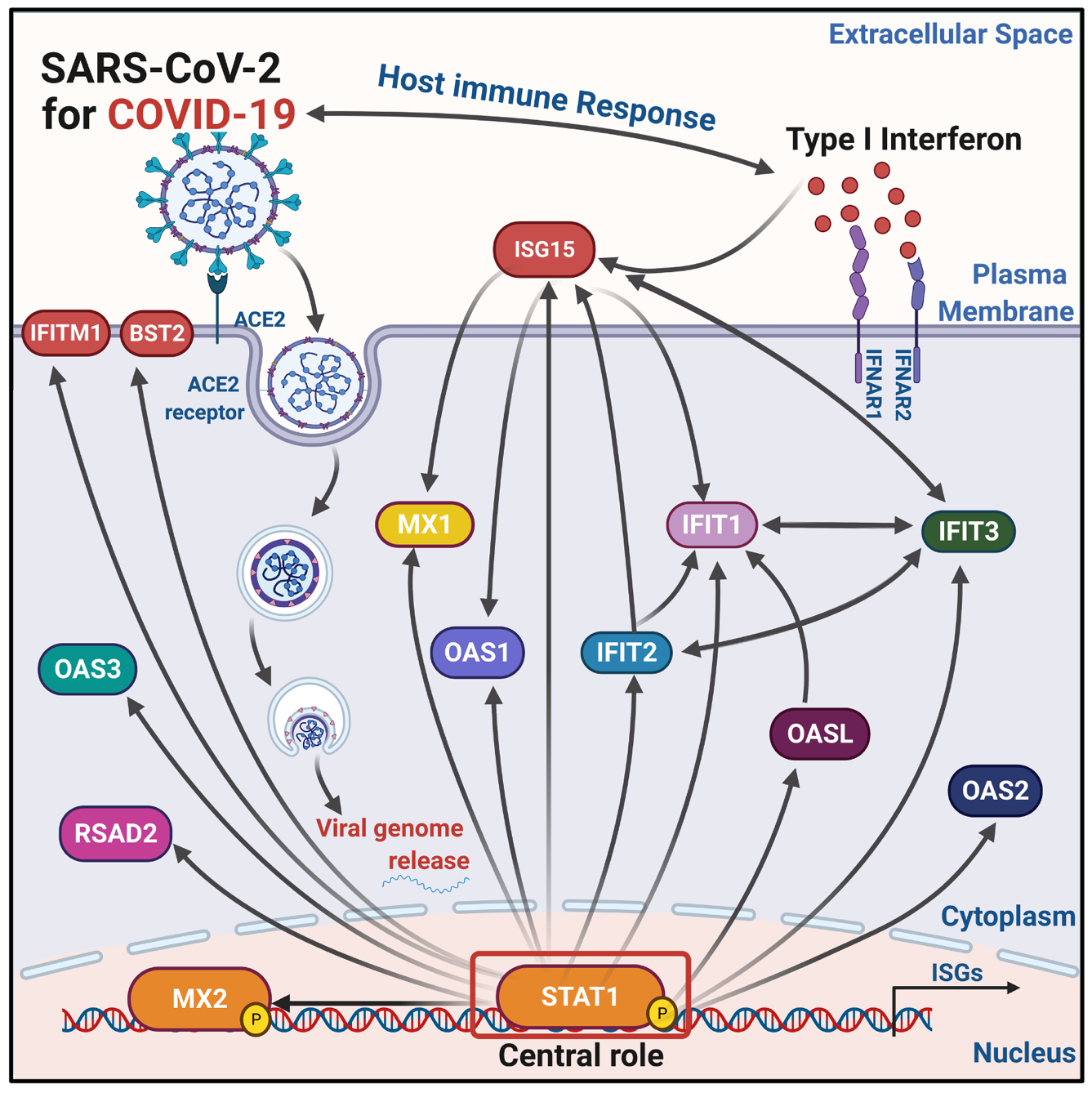
Supplementary Material 6**: Proposed schematic showing the Type I interferon-inducible host response of 14 genes in SARS-CoV-2 infection. (plotted by BioRender and Adobe Illustrator)
