# Supplementary material for: Transcriptional Profiling and Machine Learning Unveil a Concordant Biosignature of Type I Interferon-Inducible Host Response Across Nasal Swab and Pulmonary Tissue for COVID-19 Diagnosis
Source: Front Immunol. 2021 Nov 22;12:733171. doi: 10.3389/fimmu.2021.733171 (PMC8647662; doi:10.3389/fimmu.2021.733171)
Supplement: Supplementary file 10 [file DataSheet_10.docx]

**R code for analysis (****Running codes in the RStudio Version 1.3)**

# These R libraries need to be installed prior to running the code

library(WGCNA)

library("scatterplot3d")

library("rgl")

library("Rtsne")

library("UpSetR")

library("vegan")

library("GOplot")

library("fossil")

# Set the working directory for reading and saving the data

setwd("/Users/Destination/R analysis")

#=========================================================
#Data preparation #=========================================================

#read the sample data (“Supplementary Material 3-Nasal swab.txt”

or “Supplementary Material 3-Lung.txt” for WGCNA analysis)

Options(stringsAsFactors = FALSE);

expro=read.table('Supplementary Material 3- Nasal swab.txt',

sep = '\t', head=T, row.names = 1)

#Select gene expression variance greater than 90% of the whole genome

m.vars=apply(expro,1,var)

expro.upper=expro[which(m.vars>quantile(m.vars, probs = seq(0,1,0.1)[10])),]

# Convert data type to matrix

datExpr0=as.data.frame(expro.upper)

#Evaluate whether the matrix information is qualified

gsg = goodSamplesGenes(datExpr0, verbose = 3)

gsg$allOK

#optional: When GSG is not shown “allOK”

if (!gsg$allOK)
{ if (sum(!gsg$goodGenes)>0)

printFlush(paste("Removing genes:", paste(names(datExpr0)[!

gsg$goodGenes], collapse = ", ")));

if (sum(!gsg$goodSamples)>0)

printFlush(paste("Removing samples:", paste(rownames(datExpr0)[!

gsg$goodSamples], collapse = ", ")));

# Remove the offending genes and samples from the data:

datExpr0 = datExpr0[gsg$goodSamples, gsg$goodGenes]

}

#=========================================================
#Sample clustering to detect outliers #=========================================================

# All data datExpr0

sampleTree = hclust(dist(t(datExpr0)), method = "average")

# Plot the sample tree: Open a graphic output window of size 12 by 9 inches

# The user should change the dimensions if the window is too large or too small.

sizeGrWindow(20,9)

pdf (file = "HeartClustering.pdf", width = 20, height = 15)

par(cex = 1);

par(mar = c(0,4,2,0))

plot(sampleTree, main = "Sample clustering to detect outliers", sub="", xlab="",

cex.lab = 1, cex.axis = 1, cex.main = 1,lwd=1)

#=========================================================

# Choose a set of soft-thresholding powers #=========================================================

RdatExpr0 <- t(expro)

allowWGCNAThreads()

powers1=c(seq(1,11,by=1),seq(12,20,by=2))

RpowerTable=pickSoftThreshold(RdatExpr0, powerVector=powers1)[[2]]

#Plot the result, including Soft-thresholding powers and Mean Connectivity

cex1=1

par(mfrow=c(1,2),cex=1.4)

plot(RpowerTable[,1], -sign(RpowerTable[,3])*RpowerTable[,2],xlab="

Soft Threshold (power)",ylab="Scale Free Topology Model Fit,signed R^2",type="n",ylim=c(-1,1))

text(RpowerTable[,1], -sign(RpowerTable[,3])*RpowerTable[,2], labels=powers1,cex=cex1,col="red")

abline(h=0.8,col="red")

plot(RpowerTable[,1], RpowerTable[,5],xlab="Soft Threshold (power)",ylab="Mean Connectivity", type="n")

text(RpowerTable[,1], RpowerTable[,5], labels=powers1, cex=cex1,col="red")

dev.off()

#=========================================================

# Here we define the adjacency matrix using soft thresholding with beta=5

#=========================================================

beta1=5

Connectivity=softConnectivity(RdatExpr0,power=beta1)

pdf("scalefree= softConnectivity.pdf",15,10)

par(mfrow=c(1,1),cex=1.5,lwd=1)

scaleFreePlot(Connectivity, main=paste("soft threshold, power=",beta1), truncated=T,cex=1, col="red",pch=16,ylim=c(-2.5,0))

dev.off()

#=========================================================

# Convert to adjacency matrix (power)

#=========================================================

Filterdat <- t(datExpr0)

adjacency = adjacency(Filterdat, power = 5)

#=========================================================

# Convert to topological matrix and calculate dissimilarity dissTOM

#=========================================================

TOM = TOMsimilarity(adjacency)

dissTOM = 1-TOM

#=========================================================

# Gene clustering on TOM-based dissimilarity

#=========================================================

geneTree = hclust(as.dist(dissTOM), method = "average")

# Plot the resulting clustering tree (dendrogram)

sizeGrWindow(12,12)

plot(geneTree, xlab="", sub="", main = "Gene clustering on TOM-based dissimilarity",labels = FALSE, hang = 0.04);

#=========================================================

# Gene dendrogram and module colors

#=========================================================

# At least 100 genes in a module

minModuleSize = 100

dynamicMods = cutreeDynamic(dendro = geneTree, distM = dissTOM,deepSplit = 2, pamRespectsDendro =FALSE, minClusterSize = minModuleSize)

table(dynamicMods)

dynamicColors = labels2colors(dynamicMods)

table(dynamicColors)

sizeGrWindow(8,12)

plotDendroAndColors(geneTree, dynamicColors, "Dynamic Tree Cut",

dendroLabels = FALSE, hang = 0.03,

addGuide = TRUE, guideHang = 0.05,

main = "Gene dendrogram and module colors")

#=========================================================

# Calculate the eigengene, perform hierarchical clustering on the modules, and

merge the more similar modules

#=========================================================

# Calculate eigengenes

Filterdat <- t(datExpr0)

MEList = moduleEigengenes(Filterdat, colors = dynamicColors)

MEs = MEList$eigengenes

# Calculate dissimilarity of module eigengenes

MEDiss = 1-cor(MEs)

# Cluster module eigengenes

METree = hclust(as.dist(MEDiss), method = "average")

# Plot the result

sizeGrWindow(7, 6)

par(cex = 1.6, lwd=4)

plot(METree, main = "Clustering of module eigengenes",xlab = "", sub = "")

#=========================================================

# Set abline = 0.4 to merge similar modules on the cluster tree

#=========================================================

MEDissThres = 0.4

abline(h=MEDissThres, col = "red")

merge = mergeCloseModules(Filterdat, dynamicColors, cutHeight = MEDissThres, verbose = 3)

mergedColors = merge$colors

#=========================================================

# Draw a new cluster tree and module diagram with the cut module

#=========================================================

sizeGrWindow(12, 9)

plotDendroAndColors(geneTree, cbind(dynamicColors, mergedColors),

c("Dynamic Tree Cut", "Merged dynamic"),

dendroLabels = FALSE, hang = 0.03,

addGuide = TRUE, guideHang = 0.05,

cex.colorLabels = 1,cex.dendroLabels =3,cex.rowText =1 )

# Calculate the eigengenes of the merged module

MEList2 = moduleEigengenes(Filterdat, colors = mergedColors)

MEs2 <- MEList2$eigengenes

MEDiss2 = 1-cor(MEs2)

METree2 = hclust(as.dist(MEDiss2), method = "average")

# Plot the cluster tree with merged module

sizeGrWindow(7, 6)

par(cex = 1.6, lwd=4)

plot(METree2, main = "Clustering of module eigengenes",xlab = "", sub = "")

#=========================================================

# Draw a heat map based on topological overlap

#=========================================================

# Calculate the dissTOM among genes

nGenes <- ncol(t(Filterdat))

nSamples = nrow(t(Filterdat))

softPower = 5

adjacency = adjacency(Filterdat, power = 5)

TOM = TOMsimilarity(adjacency)

dissTOM = 1-TOM

adjacency2 = adjacency(Filterdat, power = 5)

TOMsimilarity(adjacency2)

dissTOM = 1-TOMsimilarity(adjacency2)

plotTOM = dissTOM^5

diag(plotTOM) = NA

geneTree = hclust(as.dist(dissTOM), method = "average")

moduleColors = mergedColors

sizeGrWindow(9,9)

TOMplot(plotTOM, geneTree, mergedColors, main = "Network heatmap plot, all genes")

#=========================================================

# Save the result of values of module Eigengenes

#=========================================================

MEList2 = moduleEigengenes(Filterdat, colors = mergedColors)[[1]]

MEs2 <- MEList2$eigengenes

colors2 <-as.character(mergedColors)

datKME<-signedKME(Filterdat, MEList2)

geneInfo0 <-data.frame(geneSymbol=rownames(t(Filterdat)),moduleColors=NEWcolor, datKME)

write.table(geneInfo0, " Supplementary Material 3.xls", sep="\t", row.names=F, quote=F)

#=========================================================

# Geometric data shown in 3D scattering

#=========================================================

cmd1=cmdscale(as.dist(dissTOM),3)

pairs(cmd1, col=as.character(moduleColors), main="MDS plot",pch=16)

par(mfrow=c(1,1), mar=c(4,3,2,3)+0.1,cex=0.85,pch=16,lwd=3)

s3d <- scatterplot3d(cmd1,color=moduleColors,angle=210,xlab="Scaling Axis 1", ylab="Scaling Axis 2", zlab="Scaling Axis 3",type = "p")

my.lm <- lm(cmd1[,3] ~ cmd1[,1] + cmd1[,2])

s3d$plane3d(my.lm,col="blue4")

#=========================================================

# Pairwise illustration of gene module correlation

#=========================================================

pairs(datKME[1:500,],

panel= function(x,y){points(x,y,col =

c("black", "blue", "brown","pink","green","turquoise","grey","red")

,pch=16,cex=1.4)

abline(lm(y~x), col='black',lwd=2.5)

text(0.5,0.8,labels = paste('R2=',round((cor(x,y))^2,2)),

col='red',cex=1)})

#=========================================================

# Pearson’s R square and P value between modules

#=========================================================

PearsonsR <-signif(cor(MEList2, use="p")

R square­<- PearsonsR^2

nSamples=nrow(datKME)

PearsonsP <-corPvalueStudent(modul,nSamples)

#=========================================================

# t-SNE analysis

#========================================================= trn <- data.matrix(cmd1)

cols <- rainbow(10)

tsne <- Rtsne(as.matrix(trn), check_duplicates = T, pca = T, theta=0.5, dims=2)

plot3d(tsne$Y, col=as.character(mergedColors),type='s',size=0.5)

#=========================================================

# UpsetR analysis

#=========================================================

all =read.table('Supplementary Material 7 for R analysis.txt', sep = '\t', head=T, row.names = 1)

listInput <- list(blackNPS=all[,1], blueNPS=all[,2], brownNPS=all[,3], greenNPS=all[,4], greyNPS=all[,5], magentaNPS=all[,6],

redNPS=all[,7], turquoiseNPS=all[,8], blue.Lung=all[,9],

brown.Lung=all[,10],green.Lung=all[,11], grey.Lung=all[,12],

red.Lung=all[,13],turquoise.Lung=all[,14],yellow.Lung=all[,15])

upset(fromList(listInput),order.by = "freq",nsets = 15,point.size = 3.5, line.size = 3.5,

text.scale = 2,mb.ratio = c(0.7, 0.3),matrix.color = "gray23",

main.bar.color = "gray23",shade.color = "#606060", shade.alpha = 0.2,

matrix.dot.alpha = 0.5, sets.bar.color = "#990000",

color.pal = 10,queries = list(list(query = intersects, params = list("grey.Lung"), color = "red4", active = T),

list(query = intersects, params = list("blueNPS"), color = "green4", active = T),

list(query = intersects, params = list("turquoise.Lung"), color = "blue4", active = T),

list(query = intersects, params = list("turquoiseNPS"), color = "tan4", active = T),

list(query = intersects, params = list("brownNPS"), color = "coral4", active = T),

list(query = intersects, params = list("red.Lung","magentaNPS"), color = "red4", active = T),

list(query = intersects, params = list("brownNPS","grey.Lung"), color = "green4", active = T),

list(query = intersects, params = list("grey.Lung","blueNPS"), color = "blue4", active = T),

list(query = intersects, params = list("grey.Lung","greenNPS"), color = "tan4", active = T),

list(query = intersects, params = list("yellow.Lung","turquoiseNPS"), color = "coral4", active = T)))

#=========================================================

# Comparative analysis for gene profile from COVID-19 samples

#=========================================================

All2 =read.table('Supplementary Material 8 for R analysis.txt', sep = '\t', head=T, row.names = 1)

GSE171668 <- apply(All2 [,1:16], 1, mean)

GSE163151 <- apply(All2 [,17:89], 1, mean)

GSE150316 <- apply(All2 [,90:94], 1, mean)

GSE147507 <- apply(All2 [,95:97], 1, mean)

GSE182569NasalSwab <- apply(All2 [,98:100], 1, mean)

GSE182569BronchioalveolarFluid <- apply(All2 [,101:103], 1, mean)

GSE162835 <- apply(All2 [,104:140], 1, mean)

MergedA <-data.frame(GSE171668,GSE163151, GSE150316, GSE147507, GSE182569NasalSwab,GSE182569BronchioalveolarFluid, GSE162835)

#Pairewise analysis between modules

pairs(MergedA[,1:6] ,upper.panel= function(x,y){points(x,y,col = c("black", "blue", "brown","pink","green","turquoise"),pch=16,cex=3.5)

abline(lm(y~x), col='red',lwd=1)}, text(1,0.8,labels = paste('R=',round((cor(x,y)),2)),col='white',cex=2))

# Pearson’s R square and P value between modules

PearsonsR1 <-signif(cor(MergedA, use="p")

R square1­<- PearsonsR1^2

nSamples1=nrow(PearsonsR1)

PearsonsP <-corPvalueStudent (PearsonsR1,nSamples1)

#Adjusted Rand index analysis

rand.index(GSE171668, GSE163151)

dist1 <-dist(GSE171668)

dist2<-dist(GSE163151)

g1 <-rclust(dist1)

g2<-rclust(dist2)

adj.rand.index(g1, g2)

# Linear regression

p <- ggplot(MergedA, aes(x = GSE171668, y = GSE163151))+

geom_point(size = 7.5,shape = 21,fill = 'blue',color = 'black')+

theme(axis.text.x=element_text(color="black", size=35),axis.text.y=element_text(color="black", size=35),

axis.title=element_text(size=18),axis.line = element_line(size=1, colour = "black"))

p1 <- p + geom_smooth(method = "lm", formula = y ~ x, size = 2,color="red3")

ggMarginal(p1,type='densigram',groupfill=T,groupColour = F,colour="black",lwd=1.3, margins="both",size=4,fill="#FFCCCC")

dev.off()

#=========================================================

# Comparative analysis for gene profile of samples from COVID-19 and other respiratory infections

#=========================================================

All3 =read.table('Supplementary Material 9 for R analysis.txt', sep = '\t', head=T, row.names = 1)

GSE171668 <- apply(All3 [,1:16], 1, mean)

GSE163151 <- apply(All3 [,17:89], 1, mean)

GSE163151InfluenzaA <- apply(All3 [,90:144], 1, mean)

GSE163151InfluenzaB <- apply(All3 [,145:159], 1, mean)

GSE163151RSV<- 163apply(All3 [,160:167], 1, mean)

GSE32155lMeasles <- apply(All3 [,168:170], 1, mean)

MergedB<- data.frame(GSE171668,GSE163151,GSE163151InfluenzaA, GSE163151InfluenzaB,GSE163151RSV,GSE32155lMeasles)

# Pairewise analysis between modules

pairs(MergedB[,1:6] ,upper.panel= function(x,y){points(x,y,col = c("black", "blue", "brown","pink","green","turquoise"),pch=16,cex=3.5)

abline(lm(y~x), col='red',lwd=1)}, text(1,0.8,labels = paste('R=',round((cor(x,y)),2)),col='white',cex=2))

# Pearson’s R square and P value between modules

PearsonsR2<-signif(cor(MergedB, use="p")

R square2­<- PearsonsR2^2

nSamples2=nrow(PearsonsR2)

PearsonsP <-corPvalueStudent (PearsonsR2,nSamples2)

# Linear regression

p <- ggplot(MergedB, aes(x = GSE171668, y = GSE32155lMeasles))+

geom_point(size = 7.5,shape = 21,fill = 'blue',color = 'black')+

theme(axis.text.x=element_text(color="black", size=35),axis.text.y=element_text(color="black", size=35),

axis.title=element_text(size=18),axis.line = element_line(size=1, colour = "black"))

p1 <- p + geom_smooth(method = "lm", formula = y ~ x, size = 2,color="red3")

ggMarginal(p1,type='densigram',groupfill=T,groupColour = F,colour="black",lwd=1.3, margins="both",size=4,fill="#FFCCCC")

dev.off()
